# Book Reviews

**Published:** 1812-11

**Authors:** 


### CRITICAL ANALYSIS
### OF RECENT PUBLICATIONS
### IN THE
### DIFFERENT BRANCHES OF PHYSIC, SURGERY, AND MEDICAL PHILOSOPHY.

*An Address to the Apothecaries of Great Britain; with an Appeal to the Committee to which the Interests of Pharmacy are delegated, by the General Meeting held at the Crown and Anchor Tavern, London, July 3d,* 1812. *By* PHARMACOPOLA VERUS. 8vo. London, 1812. pp. 67.

*A Letter on the State and Condition of Apothecaries, with Proposals for making their Office more respectable, and more beneficial to the Public. Addressed to Pharmacopola Verus, by a* TRUE SURGEON. 8vo. Lond. 1812. pp. 27.

*A Letter in Reply to Pharmacopola Verus, and to a True Surgeon, with Proposals for Parliamentary Regulation. By a* MIXED PRACTITIONER. 8vo. Lond. 1812. pp. 21.

THE attempts which within a few years have been made to reform the medical profession generally, or to establish the rights and remove the grievances of particular classes of medical men, are, probably, so much known to our readers, that a detailed statement of them here will hardly be expected. Four prominent objects have marked all these attempts: a general reform and melioration of the profession throughout all its branches, as projected and pertinaciously, perhaps meritoriously, persevered in by Dr. Harrison; the endeavor of the licentiates to procure a recognition of their alleged rights, by the College of Physicians of London; the elevation of the Surgeon's Company into a College of Surgeons; and that of the Apothecaries to restore the current of their avocation to its ancient channel, from whence it had been diverted by the cunning manœuvering, it is said, of some physicians.

Dr. Harrison, and with great justice, urges, that the municipal pandect of the medical faculty, is altogether inefficient, or so notoriously defective, that it allows, if it does not countenance, every species of empiricism; and that grocers, druggists, coblers, and carpenters, are permitted, under this motley digest, to practice on the health and lives of his Majesty's liege people. The Licentiates bitterly complain that the fellows withhold from them privileges and rights almost acknowledged, and hardly doubted; and, after being

 again again and again repulsed, meditate a return to the charge, again, perhaps, to be defeated. The Apothecaries, alarmed for their occupation, associate, harangue, and form resolutions. In the year 1812, thus stand the medical politics of the British metropolis. The fellows of the Royal College of Physicians of London, wrapt in the panoply of their Charter, dream of security in Warwick-lane, while the Licentiates are depositing, grain by grain, the powder which they fancy will disperse into thin air, the black-letter privilege and ancient authority of these "sad and learned" chiefs. Already the volcano obscurely grumbles, and the distant thunder mutters. The Apothecary, with more serious cause of complaint than the Licentiates of the College, loudly sounds his tocsin,

"And pestles peal a martial symphony."

Fellows, Licentiates, and Surgeons, are the objects of his apprehensions. Through this mortal din, the College of Surgeons, though not exempt from domestic broils, contrives to cut its way, *pro aris et focis*, keeping in view, with steady dexterity, the good things of this world.

In this state of parties, a meeting of the Apothecaries of London was convened by advertisement, for the ostensible purpose of considering the operation of an Act proposing an additional duty on glass, as far as "it affects their particular interests;" but having an interior object in view, of greater importance to them, and to society. To inquire into the actual state of the fact of the encroachment made on their occupation by certain *new men*, under the denomination of druggists; and by what means that encroachment is to be repelled, appears to be the more serious object of this association.

At the meeting of Apothecaries, thus convened, on the 3d of July, 1812, Mr. Burrows in the chair, certain resolutions were proposed and carried. But, as these resolutions bear, *primâ facie*, no other import than what relates to the price of phials, we shall no further notice them than as they are the foundation of a committee of respectable practitioners, who may reasonably be expected to suggest some method of retrieving the practice of the Apothecary.*

* This committee, elected July 3, 1812, consists of Mr. BURROWS, *Chairman;* Messrs. Simons, Nevinson, Chilvers, Brande, Walker, Tegart, Field, Upton, Hunter, Rivers, Hurlock, Wells, Ewbank, Pennington, Forbes, Douglass, Debruyn, Cates, and Drew.

All communications, relative to the objects of this Meeting, to be addressed (post paid) to Mr. Burrows, Chairman, Bloomsbury-square.

Though

Though we have always been solicitous to avoid going into any subject that relates to the trade of medicine, whether in the hands of the physician, the surgeon, or the apothecary, we consider this to be of too much importance to an ancient, respectable, and useful, branch of the medical faculty, and to society itself, to be passed over in silence.

The complaint is, that the practice of the Apothecary has passed, or is passing, to no small extent, into the hands of a description of men unknown in former times; who, being ignorant of the elements of medical science, exercise the trade, to the injury of the regular Apothecary, to the hazard always of the public, in some instances to the direct, and in many to the indirect, destruction of human life.*

If this be the fact, the great question is, how has it arisen, and how is it to be removed?

In pursuing this inquiry, it would be a remarkable fact in the history of medicine, if some regular Physicians were found almost to coalesce with the empiric, in an endeavor to injure the Apothecary. Mine is the infallible remedy, says the empiric, buy it, and you will want no Apothecary's shop; my prescription, says this class of physician, can only be compounded at a particular druggist's with correctness and certainty. This is so monstrous and so repugnant to the feelings of a man of liberal education, considered as a project to put money into the physician's pocket, and as an illusion practised on the physician's patients, that we should not give credit to it for an instant, on less authority than a fact stated by *Pharmacopola Verus.* "An event," he says, "occurred in 1807, which, had it been noticed and managed as it deserved, might have turned greatly to the advantage of the Apothecaries' cause. I allude to the dissolution of the firm of a highly celebrated house at the west end of the town, when two *respectable Members of the Royal College of Physicians, and an Apothecary, appeared in the London Ga-*

* Of this many instances might be adduced. Two of recent occurrence may at present suffice. A druggist, in the habit of dispensing, and occasionally presuming to prescribe, sent to a child a scruple of *Hydrarg. nitric. oxyd.* instead of rhubarb. The child did not take it because it was prevented by the accident of the powder being shewn to a medical practitioner. Mr. Davis, in a circumstantial account of four children, poisoned by arsenic, inserted in this Number, very clearly shews how the danger was increased by the unwary parents applying, in the first instance, to a chemist and druggist, and how the loss of life was thus, probably, occasioned.

*zette,*

*zette, as having been long united as apothecaries, druggists, and chemists.*"

This fact explains, very fully, why these physicians would have a predilection for the shop of the druggist with whom they shared the profits. We have heard, indeed, of physicians and surgeons who receive a per centage upon all prescriptions which they send to particular druggists; but we attach no credit to such reports, and require proof as damning as that above cited by *Pharmacopola Verus*, to make us believe them. And, even should it be proved that men bearing the honorable title of Physician or Surgeon, have thus combined with druggists, to empty their patients' pockets, we do not see that it would affect the high character of these bodies; but would rest with the unprincipled individuals who practised it, as an isolated fact from which no conclusion could be drawn derogatory to the reputation of the fraternity. We should just as soon expect to hear that Bailie, Halford, and Bree, among the physicians—and Cline, Cooper, and Abernethy, among the surgeons, had taken a purse on the highway, as that they had entered into such disgraceful compacts. We can suppose, however, that little jealousies toward the Apothecary may arise in the bosoms of physicians and surgeons of unsettled and doubtful reputation, which may lead them to endeavor his expulsion from the families where accident has given this description of physician or surgeon some footing. Two motives may give rise to this: the fear of submitting their practice to the inspection of an intelligent and experienced apothecary, and the desire of increasing their fees by the chance of every trifling occurrence requiring their attendance. After all, however, if it can be proved that the avocation of the Apothecary is useless and superfluous, let it be struck from the list of human employments. But this, if it ever is effected, must happen from a fair proof of its inutility, and not from the duplicity, self-interest, or low cunning, of a few individuals of other branches of the profession.

Of the three pamphlets before us we have not much to say: they are evidently written "on the spur of the occasion," and cannot be expected to possess much literary merit, or deeply argue the question. The first goes more fully into the subject than the others; the second is evidently the production of a philosophic mind, sensible of the value of determinate facts, and incontrovertible truths; it has also a greater portion of literary merit and clearness of composition. The third has some spirit, and a little flippancy.

A short extract from each will give our readers some idea of the manner in which they are written.

Of

Of the origin, and of the means that ought to have been employed, to check the inroads which the druggist has made in the occupation of the apothecary, *Pharmacopola Verus* observes,

"When it was first felt by the apothecaries that the practice of carrying prescriptions to druggists, was a sensible diminution of that business which they had been accustomed to exercise, why did they not demand an explanation, and abolition of the then incipient custom? The druggists at that time were the humble servants to the apothecaries, almost solely depending upon them for support. Veterinary medicine was then but little in vogue; the dyer had not brought his art to the present perfection and extent; nor did the brewer brew without malt or hops. At that period the intimation would have been equivalent to a command, and the monster would have been strangled in its birth. They originally owed their existence, as a separate body from the grocers, to the support and favor of the apothecaries; they have now become their rivals, and, not content with robbing them of their just profits, they actually usurp and exercise the character and functions of their patrons!

"Had the Pharmaceutical Association, which arose in 1794, confined its objects within its proper sphere, the practitioner would at this day have blessed its formation. If my memory serve me, one of their proposed obligations was, that every member should pledge himself not to employ any druggist or chemist, who dispensed medicines on his counter; but give the preference to those who confined themselves to the wholesale trade. This was an admirable suggestion, for, as the custom did not then prevail to the extent we now witness, it was not unlikely that the druggist would prefer a certainty to that which was fortuitous, and forego present profit to secure his future interest.

"Had this regulation been universally complied with by the town and country practitioners, the system would have been effectually stopped for ever.

"Although eighteen years have since passed away, and this practice has become inveterately confirmed, I do conceive, that still the recommendation and adoption of such an expedient would be attended with the happiest success, if supported by general concurrence. It may be said that the Country would never cordially join with the London practitioner in this measure, because the former are so much less affected by it than the latter. They who suppose so, know little of the real case. The quantities of medicines daily dispensed and expedited into the most distant parts of the country, exceed the credibility of every one who has not seen them as I have. Every species of pharmaceutical preparations which contain spirit or aromatics, sufficient to preserve them for a few days, are now ordinarily sent to the families of people of fortune when resident in the country. The provincial cities abound with dispensing druggists, and every market-town has at least one of those monstrous anomalies, ycleped, a practising chemist.

"In

"In the profession of the healing art this may surely be designated a new era. In the present month, we have beheld one of these non-descripts actually called upon, and delivering his opinion in a public court of justice, upon the physical and mental competency of a testator upon his death-bed to assign his property by will (vide the public papers, in the report of a cause, Warrand *v.* Wadman). Thus we behold the druggist assuming the real character of the apothecary, and the latter, urged by the impossibility of gaining a subsistence by the pursuit of regular practice, adopting the business of the former, if he fortunately possess a sufficient capital. How apposite is the hacknied quotation, '*Tempora mutantur et nos mutamur in illis.*'

"Unfortunately country practitioners are very often connected with their London druggists by the ties of obligation, for numerous little commissions which they require to have executed in town, for which they consider and constitute their druggists a species of agent. But the inconveniences they would at first experience, would soon be relieved by the selection of a new connection.

"Numbers of the most respectable druggists would immediately embrace the proffered conditions, and amply compensate themselves for the loss of their retail, by the increase of their wholesale, business. I may be too sanguine in my ideas, but I at present feel firmly persuaded, that the plan, if *now* executed, would be attended with extensive and most important benefits: unquestionably much of the business supposed to be lost, would speedily revert to its proper and original possessors.

"It would be a point at issue between the apothecary and the druggist, with which the public would have nothing to do, and in which the other branches of the profession could not openly interfere, for the sake of that consistency and decency, which, if not sincere, they are outwardly obliged to exhibit and profess."

Of the utility of the Apothecary, or rather the Surgeon-Apothecary, in society, this writer adduces many proofs: we must, however, only cite the following.

"In the pursuit of his avocations, every man of observation may enjoy opportunities of making certain just deductions from visible and acting causes that are producing important effects on political economy.

"If, as we are told, the power and strength of a nation consist in the extent of its population, it were well worth the consideration and care of the government, to view prospectively the probable tendency of the relative condition of medicine, in the United Kingdom, on this resource of our national power and proud pre-eminence.

"While disease and other casualties are the allotment of mortality, the two superior orders of the profession will flourish; they are entrenched and supported by chartered rights, while the apothecary is protected only in name. If this last order be of no service to the community, let the danger which threatens its very existence fall, and let it sink into nothingness and oblivion; but if it be really of any importance, if it possess any merits, if the country (that is, the mass of its population) cannot dispense with its medical services, why should it not be protected as an efficient and integral link in the great chain of society, without which there would be neither strength nor stability. The fact is, that they who unite the practice of pharmacy with surgery and midwifery, are indispensible for the care of the public health.

"From them are educated and trained the numerous medical assistants for the naval and military departments of the nation. They supply almost all the practitioners to our many colonies. Their practice and care constitute the nursery for young men constantly required to fill up the vacancies our active service and extended dominion occasion."

The following general observations, from the second pamphlet, seem very much to deserve the notice of the faculty, especially that branch of it educated under the surgeon-apothecary.

"The labor and anxiety of mind, the previous education, the heavy charge and responsibility of every class of medical men, render it highly just that they should be paid according to the means of their employers, and the measure of their own experience and reputation. It is also just that the health and lives of the middling class of society should be as well secured as the nature of human institutions will allow: and here I must again repeat, that the class of apothecaries must be always the managers of sickness among the majority of individuals composing civil society, and that it is equally the duty and interest of all, to be assured of their competency. This security can, in my humble opinion, only be obtained from an authoritative body, empowered to ascertain the qualifications of candidates, and to grant a license, no matter under what name, provided it gives the privilege to recover, in our courts of law, reasonable and stated charges for skill and attendance.

"The prevalent diffusion of what is termed general knowledge, has undermined the great basis of medical mystery; and it would be well for the followers of that art, to be in advance with this progressive change. I will not say that fashionable or popular lectures are calculated to give profound views, or, perhaps, to do much good; but they have put medical men upon their mettle, in such elementary subjects as anatomy, chemistry, and natural knowledge; and it now behoves us all, both for ourselves and successors, to take a fair and honorable ground, and to stand before the public, and by the side of other learned professions, on our real merits. The health and life of man is surely as valuable to him as any other concern, temporal and spiritual affairs being interwoven with both.

"It is of no consequence to the diseased, by what designation the person is called to whom his confidence is given, and his means are bestowed for his personal safety. The reward for such responsibility should be equal to that of any other profession; and it would be so, if medical men were as attentive to the real interests of their patients, patients, as they are to punctilious distinctions and worldly gain. The discreditable bickerings about college etiquette, the disgusting false boastings of some diplomatised practitioners, affecting to outstretch their colleagues, and to cure incurable diseases, together with the flat contradictions of pretended practical writers, have brought an odium on the profession, which it may be very difficult to remove; but there are, and always will be, men above the reach of such slanders, and to them belong the reformation and advancement of their art.

"Some diseases are manageable to a moral certainty, and oftentimes by obvious means consistent with plain physical principles. A clear, ready, and confident, detection of the nature and tendency of a bodily disorder, and a candid explanation of the means to be employed, will invariably gain the confidence of the sufferer, and fill him with respect for his medical attendant. If the apothecary were to be paid for his visit, he would occasionally refrain from sending drugs, and carefully direct the diet of the diseased, which is always a principal concern; he would soon gain the same regard as the prescribing physician and surgeon, and be as certainly, and as civilly, paid.

"Those who have obtained but a moderate knowledge of human nature, will quickly perceive the uncertainty of a fair requital, when the pressure of disease and danger is over, and the unsatisfactory intercourse which must continue after an affront passed upon reasonable charges. But, if the rate of attendance was established by law, it would secure an amicable understanding throughout, and prevent the exhorbitant demands of quacks and of rapacious practitioners, and become a standard for medical charges of all sorts. It is true, that most of the respectable families, who still retain the manners and feelings of our old English gentry, are, in the habit of doubling the amount of the apothecary's bill when it is paid; but this liberal custom is rapidly declining."

From these general observations, the writer goes on to particularize a plan for the melioration of the employment of the apothecary, and of defining and legalizing his claims.

"If medical men are serious in their wishes to advance the character of their profession, I would earnestly entreat them to consider this subject deeply, and, laying aside all unworthy motives, to unite in a generous and public-spirited manner, to establish their profession upon a respectable and rational footing, and to draw a line of demarcation between themselves and every species of dangerous empirics. Not but that there are, and will continue to be, quacks in all professions, and in every station of life; but in ours it is most notorious, and is so humiliating a stigma, that well-educated young men are ashamed to adopt the medical character. If a firm band of respectable apothecaries, belonging, if possible, to the company, will faithfully unite their efforts, and call upon a number of liberal and unexceptionable members of the Colleges of Physicians and Surgeons, they may without difficulty, and with little hazard of ultimate ultimate success, draw up a plan for re-modelling the Company of Apothecaries, and for a parliamentary authority to maintain their just privileges. There are good men, and evil-minded men, in all professions, and it is but fair to expect a number of steady, true, and liberal, professors in the medical art, disposed to organize and support the claims of the apothecaries. Popular favor is sure to be with them, and there is no rank in society who are not indebted to their services, and who do not continually entrust themselves and families to this class of medical men. Even a royal physician in the highest favor, and a serjeant surgeon to the king, have both kept apothecaries' shops, within the knowledge of us all.

"These intimations for the improvement of the condition of apothecaries, and for the greater security of the public, would instantly assume a practical form, if submitted to the cool discussion of experienced and honest practitioners belonging to the three branches of the art. But, as the proposal must appear indefinite without some ground-work on which the discussion may proceed, I beg leave to submit the following suggestions:

"That, at a meeting of twelve regular apothecaries, six regular physicians, and six regular surgeons, the Charter Laws and By-laws of the Royal Colleges of Physicians and Surgeons, and of the Company of Apothecaries, be read, and a day fixed, within the subsequent week, for the parties to meet again, each prepared by a due consideration of the previous documents, to appoint a sub-committee to draw up the outline of a bill for a new charter for the apothecaries, which, in addition to the present powers and offices of the Hall, shall present a plan for a Court of Examiners to grant licenses and legal authority to practise the art of the apothecary; that is, in plain terms, to legalize what has been and must ever continue, the charge and responsibility in the apothecary, of visiting and treating sick and disordered persons, and to afford him a suitable compensation in money for every attendance.

"In return for these benefits, if secured to him, the apothecary should be expected to make no charge for his medicines; and, in case of his attending persons at a distance from his own residence, I would recommend, that he might transfer his prescription to the nearest apothecary's shop, which would prove a liberal accommodation to all parties. If each visit were to be charged at the rate of only five shillings for affluent persons, and three shillings for tradesmen, it would prove a reasonable compensation for the trouble; and, as an acknowledgment for skill, and superseding, perhaps, a packet of drugs, it could not fail to please the patient.

The author of the third pamphlet, who designates himself by the odd term "Mixed Practitioner," remarks with acuteness and spirit,

"Will our modern physicians undertake all that their progenitors did, and accommodate their fees so, that their advice and medicine may be within the reach of all classes, on all occasions of disease? If not, will they lend their aid to obtain regulations, or laws, which shall place their subordinate brethren, who have passed the early part of their lives in the study and observance of disease, and exhibition of appropriate remedies, (although they may not have obtained a diploma,) in a condition to receive an adequate compensation for their professional duties, removed from the temptation of sending unnecessary medicines to their patients? by which the public will be benefited more than the apothecaries.

"Although a skilful and experienced apothecary may, and often does, render the aid of a physician unnecessary; yet, on the other hand, it is equally true that the well-informed apothecary is always willing, and often desirous, of the aid of a physician, whenever it is consistent with his patient's pecuniary circumstances; while the ignorant and uneducated is anxious to keep the physician away, lest he should detect his ignorance.

"I dilate on this head, because I agree with P———— Verus, that the 'practising chemists, and dispensing chemists,' have deprived the regular apothecary of a large portion of his proper emoluments; and because I think this has been countenanced and *promoted* by many physicians; and some have even condescended to share profits with these chemists. Let the public, therefore, be no longer deluded by an opinion, that by not employing an apothecary they are secure from excess of medicine."

As to the precise mode to be pursued for establishing the avocation of the Apothecary on a secure basis, taking it for granted (and we believe it may be proved) that it is an avocation essentially useful to society, opinions are much at variance. The writers of the pamphlets before us disagree on the subject. We are inclined to adopt the project of the "True Surgeon" and prefer a fair and candid conference of the three branches of the profession, with the view of inducing the legislature to legalize some sufficiently matured plan, which shall clearly define the claims of the Apothecary, and enable him to recover a reasonable remuneration for his skill and his time. That man, however, must be more sanguine than experience warrants, who expects the legislature will promptly adopt a plan for the melioration of the condition of the professors of pharmacy. The interests of the druggists and their partizans would be really or ideally involved, opposition would arise, and the project be lost, perhaps, in the House of Commons, unless the prime minister, for the time being, should sufficiently understand the "SALUS POPULI SUPREMA LEX," to disregard the selfishness of all parties, and give his influence and authority to the cause of public good. If such an halcyon time should appear, then may the medical faculty expect, throughout all its branches, a reform, radical and efficient, devoid of the artifices of physicians in partnership with practising chemists, and surgeons with druggists, or of apothecaries looking only to their own emolument.

*A practical*

*A Practical Treatise on the superior Efficacy of the Dolichos Pruriens, or Cowhage, internally administered in Diseases occasioned by Worms; wherein are exhibited a concise Statement of the Symptoms of the Disease, and the uncertainty of most other Vermifuges now in use. By* WILLIAM CHAMBERLAINE, Surgeon, &c. &c. &c. 10th Edition. 8vo. Lond. 1812. pp. 108.

FEW of our readers are, we presume, unacquainted with this very useful practical Treatise on the *Stizolobium*, in some of its editions. The present (the 10th) we are induced to notice for the new matter it contains, and because we think an acquaintance with its practical facts cannot be too extensively promulgated.

Prefixed to this edition is a neat "General Description of Intestinal Worms;" the species noticed are the Ascaris lumbricoides, Tænia, Ascaris vermicularis, and Trichuris. In the infinite variety of structure in animal life, nothing, perhaps, excites our astonishment and puzzles our conclusions, more than the organisation of the species of the genus Tænia. Of the seventy-seven species of this genus which have been described, it is only the two which inhabit the human intestinal canal that are the particular objects of Mr. Chamberlaine's observations. These are the *Tænia osculis marginalibus*, and the *Tænia osculis superficialibus*. The extreme length to which these vermes have grown in the human intestine, if it were not authenticated from repeated observation, would not gain belief. Boerhaave saw a tænia thirty ells in length, and another which had twenty-one thousand six hundred joints. It will be no less a matter of surprise, however, that this immense length of animated matter may exist in the intestinal canal without exciting a symptom that shall indicate its being there. We have known a young athletic man, who had invariable good health, discharge a tape-worm of many feet in length, without having had a previous indication that he nourished such an inmate. The morbid derangements that are generally produced by this genus of the *Vermes Intestina*, are of a serious nature, however; but distinguished by symptoms too well known to require us to point them out.

The following fact, related by Mr. Chamberlaine, tends to illustrate the history of intestinal worms.

"Dr. Bloch, in his inaugural Dissertation, for which he received the prize of the Royal Society of Sciences at Copenhagen, describes a worm, under the name of *Ligula*, which resembles a ribband, and is without articulations. April 27, 1805—Dr. Willan sent to me a young gentleman, eleven years of age, who had passed several worms, worms, (one on the morning of the day here mentioned, nine inches in length.) At first sight, I conjectured it to be a very large *Teres*, but upon more accurate examination, saw that it had a very different appearance, being broad and flat, like a tape-worm. It had no articulations; but at one end terminated in a flat truncated extremity, resembling the joint of a Tænia; the other end was considerably larger, thicker, and nearly shaped like the head of an eel. It was not quite dead when I examined it. Another worm, from the same patient, exactly resembled one joint of a tape-worm, except in its length, which was eight inches; this piece was without articulations, and was half an inch in breadth. I have not seen any similar to these before or since. May we be allowed to suppose a probability of these being a part of a large worm of that species which Dr. Bloch has described under the name of *Ligula?*"

An evidence of the efficacy of Dolichos pruriens as an anthelmintic, is its now being admitted into the Materia Medica of the London, Edinburgh, and Dublin, Pharmacopœias.

The five chapters into which this treatise is divided contain—1st. General description of worms. 2d. Causes and circumstances most favorable to their propagation, as debility of the organs of digestion, unwholesome diet. 3d. The more usual symptoms; similarity of the symptoms of hydrocephalus internus, with those occasioned by worms; tabes mesenterica; symptoms of worms accounted for. 4th. Cure; inefficacy of the most usual remedies; bitter cathartics; Madame Nouffer's remedy; oils; preparations of mercury; observations on the abuse of mercury in the West Indies; Anthelmia; Asclepias; Geoffrea inermis; Dolichos pruriens; after treatment. 5th. Descriptions, from various authors, of the Dolichos pruriens, and testimonials of its utility.

---

*The Principles of Physiological and Physical Science, comprehending the Ends for which Animated Beings were created, and an Examination of the Unnatural and Artificial Systems of Philosophy which now prevail. By* Richard Saumarez, Esq. 8vo. pp. 425. Murray, Egerton, &c. &c.

(Continued from p. 337.)

Chapter IX. *On the Process of Gassification.*—Having stated the various means by which factitious airs are obtained, and the opinions of different authors upon this subject, Mr. Saumarez contends, that it is to the influence of the solar rays, that the process of evaporation and of gassification, which is so extensively carried on from the surface of the globe, is principally to be referred; by which water and other liquids, owing to the minute division to which the particles of which they are composed are susceptible, become acted upon, and changed, from a liquid to a gaseous, from an inelastic to an expansible, state; leaving wet places dry, and dry places fissured and cracked; fogs and clouds undergo a transmutation from moisture to dryness, from opacity to transparency, from precipitation and fall to suspension and dissipation, &c.

In Chapter X. Section 1, the author proceeds to show, that, whenever air is situated in space, free and unconfined, this expansible power of gases is exerted equally in every direction; radiating, as it were, from a centre, it becomes extended to the whole circumference; as we behold when a small portion of air is enclosed in a large bladder, and placed under the exhausted receiver. It is owing to this particular, that every portion of air is in equilibrio with the whole; that it has as much the power of rising as of falling; that it possesses as much of levity as of apparent gravity, of ascending into the nostrils as of descending into the lungs; that we feel no weight; that, in fact, the equable pressure of the air in every direction, under the same circumstances of external influence, is no more capable of smashing our bodies to a cake, than it is of bursting the parietes, or sides of the thinnest air bubble that can be conceived.

Section 2 is on *the Equilibrium of Liquids and of Solids.* After combating the idea that the pressure of water in water, or of liquids in liquids, of equal density, is in proportion to their perpendicular weights, Mr. Saumarez maintains, that this proportion, which constitutes the fundamental principle of hydrostatic science, is altogether unfounded. Amongst the proofs which he advances, he observes, "that bodies placed at the bottom of a column of water, suffer no more pressure than when they are immediately below the upper surface of it; that, instead of suffering any alteration in figure or in form, by the increased pressure, which it is erroneously supposed they undergo, that they continue uniformly the same, in whatever portion of the column they may be placed. The most brittle bodies are often immersed at a depth the most profound, and exposed to this supposed pressure without cracking—the most flexible without bending: the finest fibril of the finest blade of grass which grows at the bottom of the ocean, is found to wave and to float, erect and rigid, under this immense column of water, without suffering more pressure from it than the grass of the field does from the medium of air in which it is involved;" and, amongst other facts equally striking, he adduces the circumstance of those divers divers who often go to the depth of fifteen fathoms; and, finally, of the hydrostatic balance itself, in which it is found that the same quantity of matter on the scale, which is suspended with air, will balance the matter in the one which is situated under the surface of the water as perfectly at the depth of fifty fathoms, as it will do at the depth of one.

In order to account for this state of equilibrium, the author maintains, "as an undeniable and universal truth, that opposing forces, equally balanced, destroy each other, wholly and totally. If, for example, four powers, of whatever description they may be, exert equal force in opposite and contrary directions, the effect of the whole is absolutely lost by the separate power of each. If equal bulks of matter, whose densities are equal, are put in opposite scales, so long as they are of equal magnitude, and of equal densities, they subsist in a state of equilibrium or of balance. In like manner, it is by the pressure of water in *every direction*, that it produces no pressure *in any particular direction*. From whence he is led to conclude, that the pressure of water in water, as well as of all fluids of the *same degree of density*, when they are immersed together, instead of pressing unequally downwards, press equally sideways and upwards."

These are points of vital importance in natural philosophy, and it would seem Mr. Saumarez is himself satisfied of it, from the labor which he has bestowed in answering all the objections which can be offered to the facts which he has advanced, and to the conclusions which he has drawn. He therefore shews how it is that water is heavy in air, and that liquids, when they are converted into solids, instead of pressing equally in every direction, press in a particular one only. He is therefore led, in chapter xi. to show how and why it is bodies change from equilibrium to difference.

Chapter XI. *On the Gravity and Levity of Solids and of Liquids.*—"If a body contains as much matter as it displaces, it remains suspended and balanced; if it contains more matter than it displaces, it falls; if less, it rises. It is not to the body alone, or to the medium alone, to which these effects are to be ascribed, but to the mutual relation which exists between the one and the other."

Whilst the author demonstrates the generation of weight, of specific or relative weight, he shews, that, without the existence of a vacuum, we can form no idea of *absolute* weight. After shewing the absurdity of such a theory, he says, "until such a nonentity can be proved to have an actual subsistence, the word *vacuum*, as typical and expressive of an idea, without any prototype or exemplar whatever belonging to it, ought to be blotted out of every dictionary dictionary, the doctrine of *absolute weight* for ever abandoned, and considered a positive absurdity." This is a bold assertion, and, if true, would go far to overturn the whole Newtonian doctrine of the attraction of gravitation. After shewing that weight and motion are different things, and that a feather and a guinea, which in air move in different directions, fall from equal heights in equal times in the exhausted receiver, Mr. Saumarez says, "the weight of a body, and the motion of a body, do not therefore altogether depend on the quantity of matter in each. If these effects arise from the relation which exists between the quality of the medium and the matter by which they are surrounded, they cannot arise from the attracting power of the earth; and, if they do not arise from the attracting power of the earth, it must necessarily follow that gravity, gravitation, or weight, is a relative, not a positive term. The question then is, by what standard is this relation to be measured? I answer, the *density* which the same bulk of matter contains, with relation to the *rarity* of the same bulk of matter which it displaces. It is the density, or tenuity, of the medium which is displaced, with relation to the density or tenuity of the body which displaces it, which determines whether a body shall descend or ascend, or remain suspended, and constitutes the standard of measure. Gravitation, therefore, properly defined, is the pressure downwards which dense bodies produce on such as are rare; and levity is the pressure upwards which rare bodies produce on such as are dense.

Chapter XII. *On the Gravity or Levity of Gases.*—Although Mr. Saumarez admits, that under certain circumstances, air has weight, he denies that it is by weight that its power ought to be estimated. It neither acts by virtue of its gravity or levity, but expands to an indefinite extent, from a centre to the whole circumference.

Chapter XIII. points out, in a most perspicuous manner, the different attributes which different bodies possess. Mr. Saumarez distinguishes the *immobility* of solid matter, from the *capacity* it has to be acted upon by an external force, and the transition which it undergoes from one place to another, which change is called *motion.* He shews, that when an external force acts upon different bodies, the change which they are made to undergo depends upon the nature of their construction. If they crack or break without yielding, they are said to be *brittle*, as glass or flint; if they yield without cracking, they are *flexible*, as lead, iron, &c.; if they return to their former state after the external force is removed, through the agency of which they had been made flexible, such bodies are said to be *elastic*, such as steel, &c. With gaseous bodies, which are essentially expansible, it is far otherwise. Mr. Saumarez observes, "Instead of requiring the agency of external pressure, in order that they may be enabled to unbend and expand,—external pressure alone is the means by which this expansive power is bounded and confined;—instead of being, like flexible and elastic bodies, naturally passive and artificially active, they are naturally active and artificially inert; they are made flexible by pressure, but are expansible without it. The instant external pressure is removed, this expansive power is immediately developed from its confinement, and displayed by its activity; spreading and dilating to the utmost limits which the imagination can conceive, it communicates motion to the mobility of different bodies, and causes pressure on all."

After clearly demonstrating the distinction which exists between an acquired and an inherent property, the author remarks, "Whilst the addition of an external force is absolutely necessary to make a flexible body become an elastic one, it is by the subtraction of an external force that gaseous bodies are enabled to expand. *Elasticity* therefore is merely *vi effecto*, a power which is derived, but which does not exist essentially; it is an excited, not an inherent, power, whose energy immediately ceases as soon as the compressing cause is removed, and the particles of which the elastic body is composed, have returned to their natural and original situation. In matter, however, which is essentially expansible, it is far otherwise; instead of *vi effecto*, it is *causa motus*, not derived from *without*, but which subsists inherently *within*; not produced by external means; it is through the resistance alone opposed by external means, that this expansive power can be suspended or suppressed."

We have deemed it right to give this extract from the book, because it not only puts the subject in a new point of view, but is the principle which the author employs in order to shew that gases, when they produce pressure on other bodies, produce it, not by a gravitating, but by an expansible, force. This principle, in its application, is one of great importance in natural philosophy, because, if it be once admitted to be true, and we know not how it can fairly be denied, it goes to overturn some of the systems in physics, which have not only for their sanction the authority of time, but of universal consent.

Having described the degrees of expansible force which gases possess, Mr. Saumarez proceeds, in the xiv. chapter, to show the relation which exists between gaseous bodies, and the resistance they are able to overcome. He combats, with with great vivacity, the 3d Newtonian law, that re-action is equal to action, and proves that Newton, when he says "that a cart draws a horse, as much as a horse draws a cart, &c. &c." has ascribed equal powers to unequal causes, confounded together inanimate with animated beings, as well as different kinds of matter whose nature and properties are altogether different—death and life, passion and action, things that are moved with those that have the power of moving, things which derive power through the medium of an external force, with those which possess it essentially and in actuality; and, finally, re-action itself with resistance.

Mr. Saumarez, after expatiating on the false analogies which the illustrious Newton has made, proves "that the action produced in consequence of re-action, can neither be equal nor greater, but that it must be less." If what the author advances on this subject be matter of fact, it appears most strange that such notions should have been entertained by so distinguished a philosopher as Newton.

Chapter XV. contains a complete developement of the principles which Mr. Saumarez has laid down, and an illustration of their application. He proceeds to detail the effects which are produced by the air when there exists an inequality of resistance in the medium by which it is surrounded. "Whenever the subtraction of external resistance takes place from one part, the expansible force of the air becomes immediately dilated and directed to the particular situation where the least resistance exists; a change of pressure consequently takes place, from equality to inequality, from *radiation* equally every where, to projection unequally somewhere." Mr. Saumarez goes on to show, "that, in consequence of the unresisting state of the upper regions of the firmament, the whole column of the atmosphere progressively decreases in density in consequence of increased dilatation; that instead of the lower strata of the atmosphere subsisting in a state of condensation from superincumbent compression,—the lower strata of the atmosphere are in a comparative state of expansion from a diminution of superincumbent pressure. So far from the lower strata of the atmosphere supporting the upper, the upper strata are rather pressed up by the expansive force of the lower; instead of a progressive and gradual sinking and condensing of the whole mass from top to bottom, there is, on the contrary, a general rising and lifting up of the whole mass from the bottom towards the top."

The author supports these propositions by facts and observations which he deems conclusive, and proceeds to shew the pressure which the *expansive* power of the air must produce duce whenever the equilibrium is destroyed. Whenever the air is abstracted out of the Magdeburg hemispheres, the expansible force of the external air presses them in the closest contact. By the same means, the glass receiver is firmly fixed to the pump plate, a bladder covering an exhausted cylinder becomes burst, and mercury forced up the unresisting, because the exhausted medium which the Torricellian tube contains.

Our limits prevent us from doing justice either to the subject or to the author, by detailing at large the facts which he has here advanced, in order to show that these effects which are produced, are produced by the pressure of *expansibility*, not by the pressure of *weight*. We shall confine ourselves to two only, because they appear decisive of the point. He placed a small cylinder, having a bladder closely tied over its top, over an exhausting pump; and, over the cylinder thus capped, a receiver; so that there were, between the receiver and the cylinder, six cubic inches of atmospherical air, weighing about 10 grains, from which the external air was entirely excluded. On exhausting the air out of the cylinder, the bladder with which it was covered, burst with as much facility, by the force of those 10 grains of air, as it does when exposed to the influence of the whole weight which it is supposed the column of atmosphere exerts on the surface of the bladder. If weight, therefore, be the cause of which the bursting of the bladder was the effect, it follows, that this effect was accomplished from the weight of the air between the cylinder and the receiver. On placing, however, a weight over the bladder, not of 10 grains but of 18 pounds, it was found capable of supporting this weight without suffering any lesion whatever. In order to prove that the weight of the air is no more concerned in elevating the mercury in the Torricellian tube than in cracking the bladder over a cylinder, Mr. Saumarez placed and immersed the bottom of a Torricellian tube in a basin of quicksilver, and placed a receiver over it. In the top of the receiver a screw was inserted, with a stop-cock attached to a bladder, which had been previously filled with common atmospherical air, as nearly as possible of the same dimensions as those of the receiver. On exhausting the air out of the receiver, the mercury in the tube immediately sunk to the same level as the mercury in the basin. By turning the stop-cock, the air which the bladder contained immediately passed into the receiver, the effects of which were rendered evident by the alteration which the mercury in the basin underwent; the mercury which before was at the bottom of the tube, at the same level level with that in the basin, immediately ascended in the exhausted and unresisting medium which the Torricellian tube contained, to the elevation of 29½ inches, being of the same level as one exposed to the open air. Mr. Saumarez repeated these experiments, by filling the bladder with different gases, whose relative weights are known to be different, with tubes of different bores, varying from ⅛th of an inch to 1 inch in diameter. On exhausting the air which the receiver contained, the mercury in all sunk to the same level as the mercury in the basin; on admitting each gas to each tube, it was uniformly found, that neither the difference in the weight of the gas, nor the difference in the bore of the tube, produced any difference whatever in the elevation of the mercury. It was found that pure hydrogen gas, which is considered 13 times lighter than atmospherical air, was able to raise the mercury from its level in the basin to 29½ inches, equally and as rapidly as either the oxygen or atmospherical air, and more especially as the carbonic acid gas, which is the most ponderable of the whole; and that the size of the tube, and quantity of mercury in it, made no difference whatever, insomuch that with two grains of hydrogen gas, he was able to raise a column of mercury weighing 30 pounds to the elevation of 29 inches. From these and other facts which the author advances, he concludes, that the elevation and variation which the mercury undergoes in the Torricellian tube, is not caused by the weight or gravity of the air, and that the weather-glass now in use, called barometer, from *βαρω μετρον*, a meter or measurer of weight, is called by a term which is irrelevant and improper; but that the term ana-plometer, from the compound word *αναπλοω μετρον*, a meter or measurer of expansibility, ought to be substituted for it.

The length of the extracts from this chapter, prevents us from pursuing the application of the principles it contains in explaining the variations which the mercury undergoes, and more especially in shewing how it is, that, when the mercury sinks, the air is more heavy, but less expansible; and when it rises, the air is more expansible, but less heavy. Mr. Saumarez has anticipated and answered every objection which he thinks can be made to the facts that he has advanced: we trust, however, that on a subject which is familiar to all, and which, if the new principles were admitted, would effect a great change in natural philosophy, that Mr. Saumarez, if in error, will yet find an opponent capable of exposing and refuting his hypothesis.

Chapter XVII. *treats of Refrigeration.*

Chapter XVIII.—Section 1, *on Calorification.*—Section 2, *on the Cause and Nature of Earthquakes.*

Chapter

Chapter XIX. *on the Decomposition of Atmospheric Matter, and the Formation of Rain.*

Chapter XX. *on Comets; or the Means by which the Matter of one System is prevented from interfering with the Matter of another.*

Chapter XXI. *on the Laws of Motion.*

Before we conclude, it is our duty to observe, that the whole of the work abounds with new principles and new explanations. The facts which the author has advanced are obvious to the senses of all, and cannot be mistaken; this new system of natural philosophy, for such we will call it, if false, will therefore admit of easy refutation. The Newtonians are more especially called upon, to answer the objections which Mr. Saumarez has made to the laws of motion;—to the existence of a vacuum, and of absolute weight;—to the impenetrability of matter; and to the uniformity which Newton supposed to exist in the original particles of matter. The natural philosopher and the physiologist, we trust, will not tamely suffer themselves to be attacked, without demonstrating the truth of their principles, and thus prove the conclusions of the author to be erroneous. For our own parts, we have endeavored to present a fair analysis of the work; and, without presuming to obtrude our own opinions, we would solicit the remarks of such of our ingenious correspondents as may have more particularly directed their attention to physiology and natural philosophy.

